# Morphological encoding in language production: Electrophysiological evidence from Mandarin Chinese compound words

**DOI:** 10.1371/journal.pone.0310816

**Published:** 2024-10-02

**Authors:** Jiaqi Wang, Niels O. Schiller, Rinus G. Verdonschot

**Affiliations:** 1 Leiden University Centre for Linguistics (LUCL), Leiden University, Leiden, the Netherlands; 2 Leiden Institute for Brain and Cognition (LIBC), Leiden University, Leiden, the Netherlands; 3 Max Planck Institute for Psycholinguistics (MPI), Nijmegen, the Netherlands; University of Florida, UNITED STATES OF AMERICA

## Abstract

This study investigates the role of morphology during speech planning in Mandarin Chinese. In a long-lag priming experiment, thirty-two Mandarin Chinese native speakers were asked to name target pictures (e.g., “山” /shan1/ "mountain"). The design involved pictures referring to morpheme-related compound words (e.g., “山羊” /shan1yang2/ "goat") sharing a morpheme with the first (e.g., “山” /shan1/ "mountain") or the second position of the targets (e.g., 脑 /nao3/ “brain” with prime电脑 /dian4nao3/ “computer”), as well as unrelated control items. Behavioral and electrophysiological data were collected. Interestingly, the behavioral results went against earlier findings in Indo-European languages, showing that the target picture naming was not facilitated by morphologically related primes. This suggests no morphological priming for individual constituents in producing Mandarin Chinese disyllabic compound words. However, targets in the morpheme-related word condition did elicit a reduced N400 compared with targets in the morpheme-unrelated condition for the first position overlap in the ERP analyses but not for the second, suggesting automatic activation of the first individual constituent in noun compound production. Implications of these findings are discussed.

## Introduction

Human communication involves a multitude of distinct representations to convey our thoughts effectively. These representations are thought to reside within our mental lexicon. The mental lexicon is often described as a cognitive dictionary and refers to the part of our language system that hosts the lexical forms we know and their corresponding meanings.

Language processing theories contain assumptions about the organization and representation of words in the mental lexicon. For example, language production theories typically describe a sequence of cognitive processes encompassing various information types, such as conceptual preparation, lexical access, phonological processing, and articulation [1–3]. The intended object’s conceptual representation becomes active when engaged in speech production, for instance, when naming a picture. This activation then extends to the lexical representations associated with these concepts. Subsequently, phonological information is retrieved, encompassing word form encoding, which is ultimately utilized for articulation by triggering the relevant gestural sequences. In speech production, morphological encoding concerns activating the words’ constituents at the lexical-morphological level. Morphemes are the minimal units that convey meaning, and many studies have substantiated the involvement of morphology in speech production [1–3]. For instance, in the word production theory by Levelt and colleagues [3], morphology is the first stage of word-form encoding.

In Chinese, compounds are morphemes (constituents) combinations with compounding, the most common method for constructing new words. Unlike classical Chinese, which is primarily monosyllabic, modern Chinese, specifically Mandarin, has increased the abundance of compound words, where multiple characters (morphemes) come together to form a word with a specific meaning [4–6]. The morphological processes of Mandarin Chinese are characterized by the dominance of compounding and the effective lack of derivational and inflectional morphology, which are abundantly present in most Indo-European languages [4–6]. For example, disyllabic compounds comprise about 73.6% by type and 34.3% by token in a large text corpus [7]. Some constituents cannot meaningfully stand alone in these compounds and could be considered bound morphemes. For example, in 骆驼 /luo4tuo2/ “camel,” the individual morphemes (e.g., 骆 and 驼) are meaningless when presented in isolation, although they can be part of other compounds (e.g., 驼背 /tuo2/bei4 “hunchback”). However, for a word like 山羊 /shan1yang2/ “goat,” the individual constituents are free-standing morphemes (e.g. 山 /shan1/ “mountain” and 羊 /yang2/ “sheep”) and can be used in a sentence (e.g., 这个山很高 /zhe4ge4/ /shan1/ /hen3/ /gao1/ “this mountain is very tall”). Note that some morphemes have become “special cases,” such as 子 /zi3/, which, when free-standing, means “child” but has also become a commonly used suffix to form (concrete) nouns, such as 椅子 /yi3zi5/ “chair” or 帽子 /mao4zi5/ “hat”.

One other potentially important aspect to consider for (literate) Chinese is that due to the make-up of the Chinese writing system, literate Chinese may have some additional awareness of the meaning of the individual constituents. As English readers need to convert sound into meaning (e.g., sound → meaning), the opposite route most likely takes place for Chinese (e.g., meaning → sound), and each morpheme is typically one character (i.e., they are visually marked). This does not mean that English speakers do not realize the meaning of individual constituents, especially for transparent compounds (e.g., they would certainly be aware of “birdhouse” = bird + house). Still, a difference between the languages becomes apparent at an infrequent English (old loan) word such as “xylophone.” This would likely not be parsed in “wood” + “sound” as many might not know that the sounds “xylo”and “phone” (which originally stem from Greek) bear those specific meanings. However, in Chinese, “xylophone” 木琴 /mu4qin2/, despite being infrequent as well, due to the logographic nature of the script, would still clearly signify the free morpheme木 /mu4/ “wood.” Less obvious transparent compounds might still elicit their individual constituents more strongly in Chinese; for example, “microwave” (微波 /wei1bo1/ “microwave,” which is “tiny” + “wave”) as the script visually marks the individual constituents (e.g., 波 /bo1/ “wave”).

Therefore, the question arises of how Mandarin compound words are stored in the mental lexicon as Mandarin Chinese’s morphological makeup differs from that of Indo-European languages. There are two alternative hypotheses. One is the *full-form hypothesis* (or full-listing hypothesis), which assumes that only whole-word forms are represented in the lexicon and that a word’s derivational history plays no role in lexical access [8–10]. The alternative view, the *decomposition hypothesis*, assumes at least one level of lexical representation at which compounds are represented in terms of their constituent lexical morphemes [3].

### Full-listing hypothesis

The issue of compound representation has previously been studied by manipulating the frequency of compound words and their morphemes. The word frequency effect is one of the most robust findings in picture naming: producing an infrequent word (such as “eclipse”) is substantially slower than producing a frequent word (such as “table”) [3]. Janssen and colleagues [10] asked people to name pictures (referring to compound words). They manipulated the frequency of the compound word and its constituents using three conditions: *HH*, *LH*, and *LL* (where the first letter refers to high (H) or low (L) whole word frequency and the second letter to high (H) or low (L) constituent frequency). For example, 山羊/shan1yang2/ “goat” would be a low-frequency word, but its constituents 山 /shan1/ “mountain” and 羊 /yang2/ “sheep” are both high-frequency morphemes. They found that only whole word frequency influenced naming latencies, and hence, they proposed that their findings support the full-listing hypothesis.

Additional support comes from Bi and colleagues [11], who investigated two aphasic patients with difficulty in lexical access for oral and written production, respectively. They showed that the frequencies of the compound words themselves, but not the frequency of the constituent morphemes, affected the production performance of both patients.

In another study by Chen and Chen [12], using the implicit priming task [13] Mandarin Chinese participants named compound words in the context of a response-association task to investigate whether morphological encoding is involved in producing Chinese disyllabic transparent compound words. Their results revealed, contrasting with findings in Dutch, that naming latencies were not sensitive to the compound’s morpheme frequency. This supports the assumption that word frequency is related to word forms and the interpretation of a single-stage model of lexical access. These findings align with the hypotheses proposing the existence of a single lexical level during word production and positing that compounds possess distinct lexical nodes within this level [1]. They pose a challenge to the viewpoint presented by theories assuming morphological decomposition [3].

### Decomposition hypothesis

Some studies also present results in favor of the decomposition model. In Roelofs’s paper [14], the issue of whether the form lexicon underlying speech production contains morphologically decomposed entries was addressed by manipulating word frequency. Only decomposed form entries would allow morphemes to be planning units in speech production. The outcome supported the idea that component morphemes of Dutch compound words were planning units in speech production and confirmed the decomposition assumption. Both high-frequency and low-frequency morphemic constituents yielded a facilitatory effect. Similarly, in a study by Bien and colleagues [15], four experiments were conducted to investigate the role of frequency information in compound production by independently varying the frequencies of the first and second constituents and the compound itself. The results of these experiments revealed that compound word production was sensitive to cumulative morpheme frequency, which supports the decomposition hypothesis.

Furthermore, Chen & Chen [16] investigated phonological planning in Mandarin spoken production, focusing on Mandarin words with two characters (e.g., 玻璃 /bo1li2/ “glass”). All their experiments used the form preparation task with a slight twist. Instead of learning and producing word pairs (e.g. seeing the word 梳子 /shu1zi5/ “comb” then responding with 头发 /tou2fa3/ “hair”) which is typical in this paradigm, participants had to respond with the second constituent of a word (i.e. when encountering 玻 (/bo1/) they had to respond with 璃 (/li2/). Their idea was that when the domain of phonological planning is the entire word, the planning must always begin at the start of the word. However, phonological planning might also be influenced by the overlapping beginning of a non-initial morpheme when the domain is the morpheme. Indeed, Chen & Chen observed significant preparation effects when the targets (i.e., the second part of the disyllabic words) shared the same atonal syllable in homogenous groups (e.g., 玻璃 /bo1li2/ “glass”– 茉莉 /mo4li4/ “jasmine”– 霹雳 /pi1li4/ “thunderbolt” and 淅瀝 /xi1li4/ “pattering”). In other words, the data of Chen & Chen suggested that Mandarin speakers could plan directly from the second morpheme. Although this task is, admittingly, influenced by the experimental environment, it does support the decomposition hypothesis, as the overlap in the second constituent provided the facilitatory effect (something the full-listing hypothesis would have trouble explaining). Therefore, there still seems to be an ongoing debate on whether (Chinese) compound words are stored in our mental lexicon in a decomposed or full-listing manner.

### Long-lag paradigm

The studies investigating the representation of compounds mostly use paradigms without inherent lag, such as the implicit priming or form preparation paradigm, which may also involve activation from semantic or phonological encoding. Conversely, the long-lag priming paradigm is established and robust, especially focusing on the morphological level of representation that has proven to yield consistent and replicable results across experimental methods (i.e., behavioral, electrophysiological, and hemodynamic) and languages [17]. For instance, Zwitserlood and colleagues [18] used the long-lag paradigm to investigate the morphological priming effect. They demonstrated that morphological priming, where the prime is morphologically related to the target, remains effective despite many intervening trials. However, effects related to semantic and phonological priming were no longer observable at large lags. These findings imply that priming in those instances does not occur at a phonological or semantic level but at a distinct morphological level. This was subsequently corroborated by further investigations [19–24].

### Electrophysiological evidence

Most of the previous investigations on compound production used behavioral measures (with some notable exceptions) as mentioned above; it is therefore still unclear how the precise neuro correlates of morphological processing are manifested. Unlike reaction times (RTs), event-related potentials (ERPs) offer a higher temporal resolution that enables more direct observation of cognitive processes, even before or without an explicit response [25]. ERPs have proven useful in testing or confirming decomposition processes of compound comprehension in the visual and auditory modalities [26–28]. An ERP study investigated the effects of morphological decomposition in word reading and concluded that the N400 was sensitive to lexical status and morphological decomposition [29]. Therefore, ERPs can serve as a direct means to investigate whether morphological priming originates at the word form level, allowing for a detailed exploration of the temporal dynamics of morphological priming.

The process of morphological decomposition significantly impacts the N400 component within the context of event-related potentials (ERPs), which is closely linked to the ease of integrating words at the lexical-semantic level. This concept was initially proposed and subsequently confirmed in the field of language comprehension. Numerous studies have since then investigated this phenomenon [30–32]. A pronounced N400 component becomes apparent when participants are presented with unexpected stimuli [33]. When a target item is primed, it subconsciously prepares participants for upcoming content, leading to fewer surprises than an unprimed target. Consequently, an anticipated reduction in the N400 peak’s amplitude occurs. In language production, Koester and Schiller [21] examined the temporal aspects of morphological encoding in a picture naming task with Dutch compound words as stimuli. Their findings indicated a connection between the N400 component and morphological encoding. Moreover, a comprehensive meta-analytic review of the existing literature about twenty years ago proposed the existence of a morphological encoding process approximately 330 ms after the onset of a picture, assuming an average latency of 600 ms for picture naming [34]. Note that the time window of word form encoding could range between 217 and 530 ms (and likely even beyond that) due to uncertainties in their temporal estimates, as Indefrey and Levelt [34] pointed out.

### The position effect

Chinese disyllabic compounds have two constituents, and each constituent is either in the first or the second position. The question of whether the position will influence the decomposition process has been offered. There are two related claims. The first claim is based on the serial planning assumption, which suggests that the noninitial morphemes of a word cannot be planned before initial ones. This might lead to the expectation of larger priming effects for initial constituents [35, 36]. Additionally, the fact that modifier and head constituents (first and second constituents) are not processed alike could also affect the priming effect for two different positions. For intense, Bien et al. [15], investigated how different variables of the modifier and the head constituents affected the naming latencies of Dutch compounds. The results showed that all relevant modifier variables had facilitative effects, whereas facilitative and inhibitory effects were found for the head constituents.

Another claim can be found in Zwitserlood and colleagues’ work [18]. Their study showed that the position of the target morpheme does not influence morphological priming–both the first and second constituent of compounds cause faster-naming latencies in morphologically related targets. In addition, in Koester and Schiller’s paper [21], the authors proposed that position may play a role, but a position effect could not be straightened out from their experiment, as they used two sets of stimuli with unbalanced overlap rates. Further research is therefore warranted to clear up the existence of any potential position effect. Hence, the present study controlled the first and second positions to investigate this matter further.

### The goal of the present study

Given the situation laid out in the previous paragraphs, the present study designed a morpheme-related compound prime (e.g., 山羊 /shan1yang2/ "goat") and a morpheme-unrelated compound prime (e.g., 飞机 /fei2ji1/ "airplane") for a monomorphemic target. The target could overlap with the related prime in either the first (e.g., target 山 /shan1/ "mountain" with prime 山羊 /shan1yang2/ "goat") or second (e.g., 包 /bao1/ "bag” with prime面包 /mian4bao1/ “bread”) position. Both behavioral and electrophysiological data were collected using a long-lag priming paradigm. Faster RTs were expected in the morphologically related priming conditions [37]. A reduced N400 amplitude is expected for the morphologically related priming conditions [29]. The reduced N400 amplitude (here reflecting morphological encoding) is expected to begin around 330 ms after picture onset, assuming an average naming latency of 600 ms [34]. For the variables of position, we expected that the priming effect for the first position should be larger than the second position, given the serial planning assumption and the modifier facilitation effect in naming latencies [15, 35, 36] contrasting with Zwitserlood and colleague’s work [18]. The N400 voltage is reduced for the first position in the same time window as less cognitive effort is exerted for retrieving the first constituents.

## Methodology

### Participants

We recruited thirty-six right-handed native Chinese participants (twenty-one females) from Leiden University (Netherlands), who all graduated at least with a bachelor’s degree in China. All participants received monetary compensation for their participation. The mean age of participants was 26.6 ± 3.2 years. This study was approved by the Faculties of Humanities and Archaeology ethics committee at Leiden University (acceptance number: 2022/09). Recruitment took place between September 2022 and November 2022.

Thirty-four participants were from regions where Mandarin is spoken, including provinces of north-eastern China, north-western China, and south-western China. Two participants were native Mandarin/Cantonese bilinguals.

At the time of testing, none of the participants reported color blindness, learning disorders, hearing or visual impairments, or psychological or neurological impairments. Participants read the information sheet and signed an informed consent form before participating.

### Materials

Forty target pictures (all monosyllabic Chinese words) were chosen, and each was paired with one morpheme-related disyllabic Chinese compound word (picture) and one morpheme-unrelated disyllabic Chinese compound word (picture) as primes. Therefore, 120 black-and-white line drawings corresponding to target pictures, morpheme-related, and morpheme-unrelated prime pictures were presented as stimuli. All compounds in the stimuli list were nouns.

Seven or eight pictures, including fillers, targets, and primes, were put between target pictures and prime pictures for each pair (one target picture was paired with either one morpheme-related prime picture or one morpheme-unrelated prime picture) as intervening trials. We included 124 Chinese disyllabic filler pictures and 28 monosyllabic filler pictures to allow for the precise creation of the intervening trials. Four conditions were created in the present experiment (see Table 1).

**Table 1:** Example of a target picture with four prime types.

Prime type	Example (Prime)	Example (Target)
Morpheme-related / first position	山羊 /shan1yang2/ goat	山 /shan1/ mountain
Morpheme-unrelated/first position	飞机 /fei1ji1/ airplane	
Morpheme-related/second position	风车 /feng1che1/ windmill	车 /che1/ vehicle
Morpheme-unrelated/second position	羽毛 /yu3mao2/ feather	

Each target picture was presented twice: once with a morpheme-related prime picture pair and another time with a morpheme-unrelated prime picture pair; therefore, 464 trials were administered during the experiment. The word frequency for the four conditions was controlled based on the Zipf values from the SUBTLEX-CH corpus [38] (*F* (3,76) = 1.392, *p* > 0.05) as well as the number of strokes (*F* (3,76) < 1, *p* > 0.05) (see Table 2).

**Table 2:** Mean and SD of the number of strokes and word frequency (Zipf values) for prime types.

Prime type	Strokes		Word Frequency (Zipf)	
	Mean	SD	Mean	SD
**Morpheme-related / first position**	16.55	4.174	2.242	0.771
**Morpheme-related / second position**	16.40	4.358	2.181	0.785
**Morpheme-unrelated / first position**	18.60	5.519	2.476	0.652
**Morpheme-unrelated / second position**	15.10	4.866	2.346	0.427

Before the experiment, we tested whether stimuli have similar visual complexity (*F* (3,76) < 1, *p* > 0.05). Prime and target pairs were phonologically and orthographically unrelated. S1 Table presents an overview of all prime-target combinations used in the experiment.

### Design

A two-factorial within-subject design was adopted in this experiment, with Morpheme Relatedness (Related/Unrelated) and Position (First/Second) as the two main factors. Four conditions were created.

Two sets of main stimulus lists were prepared, and within each set were two sub-lists. Each sub-list had 232 different trials, which contained 20 primes in the related condition and 20 primes in the unrelated condition, along with 40 targets and 152 fillers. The first sub-list includes one-half of the related and unrelated primes, while the second contains the other half. Both sub-lists share the same 40 targets and 152 fillers. Each participant saw all the target and filler pictures twice and all prime pictures once, resulting in 464 (232*2) trials per person in the present study. Furthermore, intervening trials contained no phonologically or semantically related items to the target pictures.

Each sub-list was divided into four sub-block groups; hence, there were 58 trials in each sub-block. In total, eight of these sub-blocks were created for the present experiment. There were breaks among sub-blocks. Half of the participants were given the first stimulus list set during the experiment, while the other half received the second set. This approach was taken to balance the experiment and eliminate potential biases.

Due to Chinese people tending to use disyllabic words to name monosyllabic words, we used red and green frames to indicate how many words should be named in our experiment. Red-framed pictures are for monosyllabic targets and fillers, and green-framed pictures are for disyllabic primes and fillers. Because all primes (in both morphological-related and morphological-unrelated conditions) were two-character words, all prime pictures would be in green frames. Therefore, the potential effect that might be caused by different cues was counterbalanced across two prime conditions. The same goes for the target, where all target pictures were in red frames. Therefore, for two different positions, the color effect was counterbalanced as well.

### Procedure

The experiment was designed and controlled using *E-prime 3*.*0* (Psychology Software Tools) and was conducted in a soundproof booth. The procedure was similar to Koester and Schiller’s [21], adapted from Dohmes et al. [37]. First, participants were given 10–15 minutes to familiarize themselves with the target and prime picture names by studying a booklet. Subsequently, the experimenter assessed whether participants correctly remembered the picture names by conducting a practice session through E-prime. In this session, participants were asked to name all the target and prime pictures as soon as possible, and the experimenter would correct them if they named those pictures erroneously.

In the experiment session, each trial began with a fixation cross displayed for 250 ms, followed by a blank screen for another 250 ms. Then, the stimulus appeared in the center of the screen for 2,000 ms. Therefore, each trial lasted 2500 ms, which was applied to all pictures, including primes, targets, and fillers. Because participants were given color cues while naming the pictures, the stimulus presentation time was longer compared to several earlier studies [21, 23].

**Fig 1:**
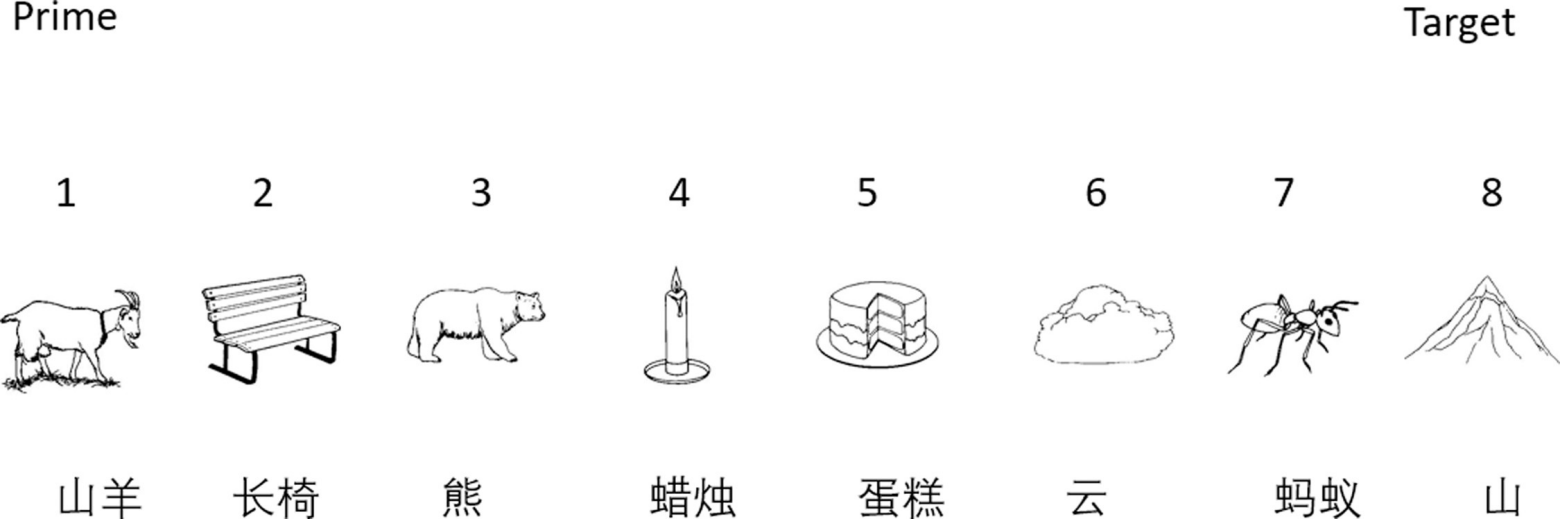
Example sequence of a prime-target combination in morpheme-related condition.

Subsequently, the experimenter recorded the validity of each trial by noting target language errors, word errors, and voice-key errors. No feedback was provided during the experiment. Throughout all three sessions, target pictures and monosyllabic filler pictures were displayed in a red frame, while prime pictures and disyllabic filler pictures were shown in a green frame. We used a long-lag paradigm for the actual experiment session. Fig 1 illustrates how stimuli were presented in this long-lag paradigm.

Additionally, we presented two sub-lists to each participant during data collection. To counterbalance the order effect, we alternated the sub-list presentation order: Participant 1 saw sub-list 1 first and then sub-list 2, Participant 2 saw sub-list 2 first and then sub-list 1, and so on. Moreover, we had two versions of the main stimuli. Half of the participants used the first version, and the other half used the second version to minimize order and repetition effects.

### Electrophysiological recording and data processing

EEG data were collected using *BrainVision Recorder* software (*Version 1*.*23*.*0001*) by *Brain Products GmbH*, with an *EasyCap* electrode cap configured according to the standard 10/20 montage (see S1 Fig). The recordings utilized 32 electrodes (*BioSemi ActiveTwo*) positioned on the scalp according to the standards of the American Electroencephalographic Society (1991). Additionally, the vertical electrooculogram (*VEOG*) was recorded using two external electrodes placed above and below the participant’s left eye. In contrast, the horizontal electrooculogram (*HEOG*) was recorded with electrodes positioned at the outer canthus of each eye. Two flat electrodes were attached to the mastoids. CMS and DRL electrodes served as ground references. The EEG signals were subsequently re-referenced offline to the mean of the two mastoids. Data were sampled at 512 Hz, and an offline band-pass filter ranging from 0.01 to 30 Hz was applied, following established procedures from previous studies [21, 24].

### Data analysis

#### Behavioral and EEG data exclusion

The two bilingual participants’ naming latencies and EEG data were excluded because their native languages were Cantonese and Mandarin. The naming latencies of the other two participants were excluded because of their low accuracy rate. The EEG data for the four other participants were lost due to EEG recording failures and insufficient signal quality. Therefore, thirty-two participants remained for naming latencies analysis, and twenty-eight remained for EEG data analysis in the present paper. For the remaining data, 8.32% of data trials were excluded from further RT analysis, including error trials and trials with reaction times, which deviated more than 2.5 SDs from the mean per participant per condition. For EEG data, 23.15% of data trials were eliminated from the ERP data analysis, including error trials (7.99%) and epochs removed during artifact rejection (15.16%).

#### Behavioral data analysis

Behavioral data were analyzed using *RStudio Version 4*.*2*.*2*. Initially, we calculated descriptive statistics for naming latencies for each condition (see Table 3). Subsequently, a single-trial modeling approach was implemented using the lme4 package [39]. Specifically, we employed a generalized linear mixed-effects model (*GLMM*) with the *glmer ()* function, utilizing a gamma distribution to appropriately model the positively skewed reaction time (RT) data.

**Table 3:** Mean naming latencies (only correct trials included) for each condition.

Condition	Naming latency (ms)	
	Mean	SD
Morpheme-related / first position	779	213
Morpheme-related / second position	809	212
Morpheme-unrelated / first position	776	202
Morpheme-unrelated / second position	798	203

Random effects were chosen in such a way as to avoid over-parameterization and to balance Type-I error and power [40]. We followed a modeling approach where we maintained the simplest possible model structure in light of our main manipulations [39–41]. Our model included *relatedness* and *position* as fixed factors, *prime semantic category*, *target semantic category*, *prime animacy*, and *target animacy* as co-variates. As participants saw the targets twice, we also included the *sequence* of targets as a co-variate. *Subject* and *item* were the two random effects in the model.

We performed model fit checks by plotting the model residuals against predicted values. We used the *avova ()* function to perform model comparisons and likelihood ratio tests based on Akaike’s Information Criterion, AIC [42], BIC [43], and the log-likelihood to establish the best-fitting model for our data. When applicable, we performed *Tukey* corrected post-hoc contrasts to estimate effect sizes using the *emmeans* () function [44].

**Fig 2:**
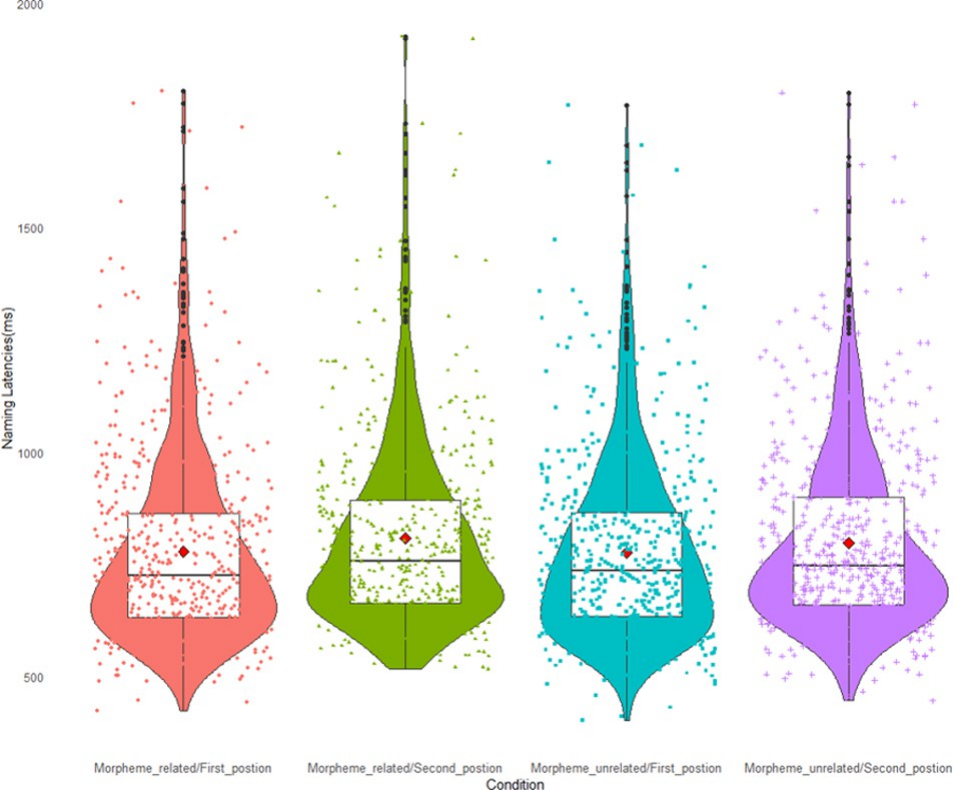
Mean naming latencies by condition for the picture naming task (n = 32).

For naming latencies, the model of the best fit was *RT ~ relatedness + position + sequence + (1|subject) + (1|item)* (see S2 Table). The Subject and Item’s random slope could not make the model converge, so they were not included in the current mode. When doing the data analysis, we first introduced the interaction of the relatedness and position in our behavioral data analysis. However, the interaction between relatedness and position did not significantly improve the model after the ANOVA test (with *p* > 0.05) compared to the model without interaction, so we decided to exclude it from the behavioral model. Contrary to findings in other studies, participants were not significantly faster in naming morpheme-related items compared to morphologically unrelated items with *β* = 0.012, *SE* = 0.010, *t* = 1.159, *p* = 0.247 (Fig 2). Position yielded no significant effect either (*β* = -0.051, *SE* = 0.045, *t* = -1.140, *p* = 0.254). The final model included the factor sequence, showing a significant effect (*β* = -0.059, *SE* = 0.010, *t* = 5.69, *p* < 0.001). The model did not include the target animacy, prime animacy, target categories, and prime category co-variates due to non-convergence or singular fit or because they did not fit the model significantly.

#### EEG data analysis

EEG data were pre-processed using *Brain Vision Analyzer 2*.*1 (Brain Products GmbH)*. We followed the EEG data preprocessing procedure laid out by Von Grebmer zu Wolfsthurn and colleagues [48]. The process included visual inspection of the signal, re-referencing, and linear derivation for the HEOG and VEOG electrodes, followed by a band-pass filter with a low-pass cutoff at 0.01 Hz and a high-pass cutoff at 30 Hz. Ocular correction and artifact rejection were then performed. Offline re-referencing was conducted to the average of the left and right mastoid electrodes. Subsequently, epochs of corrected trials were generated around stimulus onsets, and baseline correction for each segment was performed using the average EEG activity from the 200 ms preceding stimulus onset [45].

**Fig 3:**
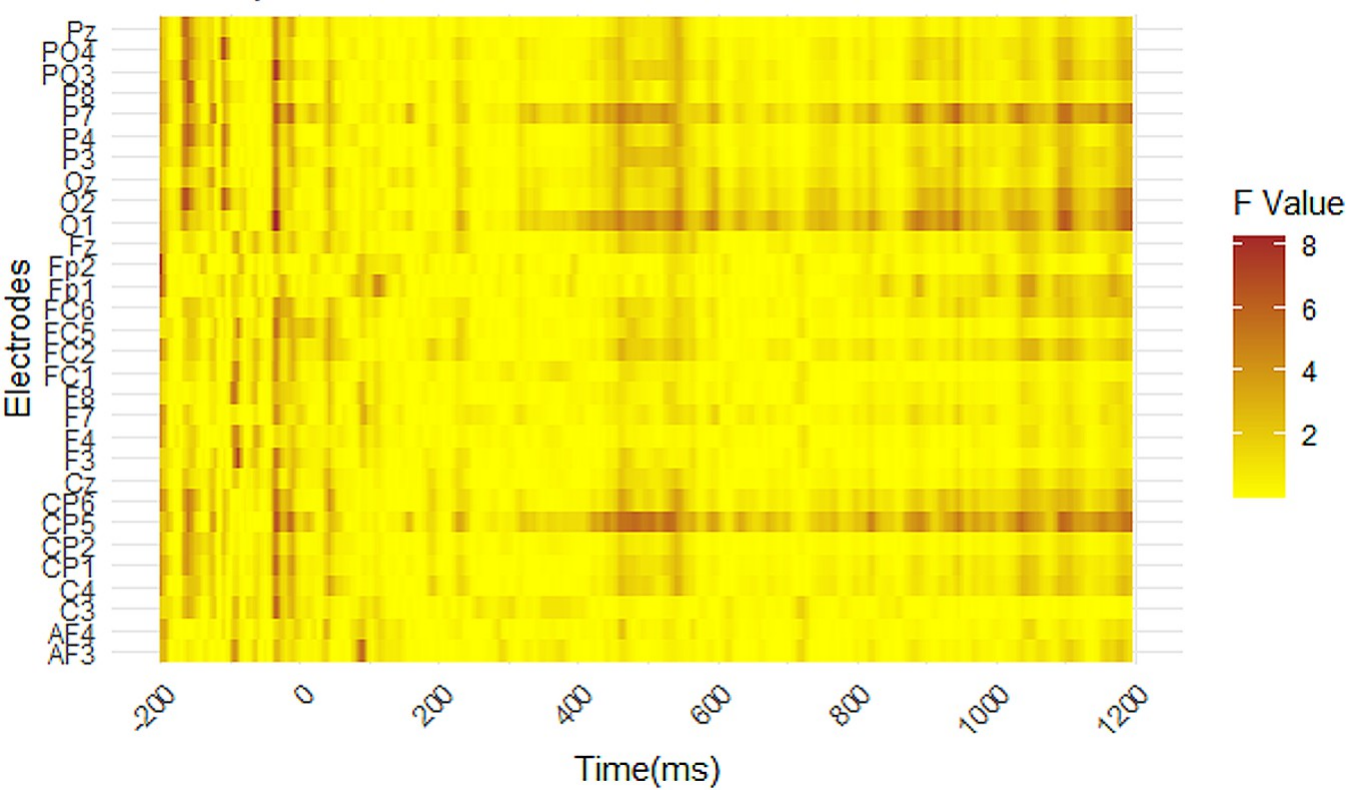
Permutation tests across all electrodes. The time window is between 0 and 800 ms post-stimulus onset. Larger F-values are depicted in darker colors, indicating an increased likelihood of a statistically significant effect of our manipulations on voltage amplitudes.

After pre-processing and exporting our data, we conducted a cluster-based analysis to tentatively explore regions of interest and potential time windows associated with significant modulations of the EEG signal. We used a permutation test computed with the *permutes* package (Voeten 2019), which included the voltage amplitudes for all data electrodes across the time window from 0 ms to 800 ms across conditions. This analysis was performed before considering the mean naming latencies of four conditions to minimize the influence of artifacts during data analysis [45, 46]. As shown in Fig 3, the output of this test indicated potentially significant modulations of the EEG signal within the time window between 415 ms and 575 ms. This time window has been previously associated with the N400 component.

Next, we divided electrodes into six areas of interest, i.e., anterior-left, anterior-right, central-left, central-right, posterior-left, and posterior-right regions [23]. Based on the output from the permutation test and previous literature associating central and posterior regions to the N400, we defined our ROIs as the following electrodes: *P3*, *P7*, *CP5*, *O1*, *C4*, *FC2*, *FC6*, and *Fz* (see S1 Fig).

Finally, we employed a single-trial linear mixed models (LMM) approach [47] to enhance the traditional average-type analysis. The traditional method has been criticized for its limitations, including equally weighted observations by condition, participant, and independent factor levels [45, 46]. These assumptions are often compromised due to design factors and during the EEG data pre-processing stages [45, 46]. An alternative method is single-trial linear LMM, which has been endorsed by many researchers since its first application to EEG data in 201 [48]. This model includes fixed effects and estimates the random variance between subjects and items, known as random effects. It can be applied to datasets with variable effect sizes and unbalanced designs [49, 50].

**Fig 4:**
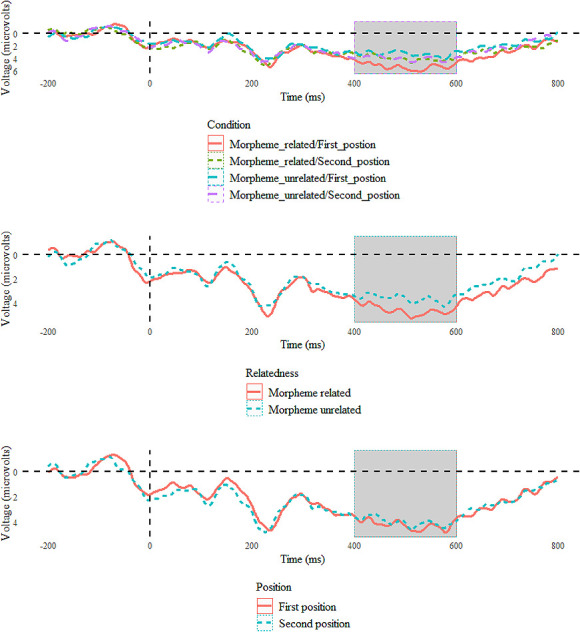
Voltage amplitudes by condition, relatedness, and position over time. For channels C4, FC2, FC6, Fz, P3, P7, CP5, and O1 for the picture naming task (n = 28), the time window of the interest is from 415ms - 575ms and is highlighted in grey. Negativity is plotted up.

For the single-trial LMM approach, we incorporated all available voltage values for each epoch within the designated time window of 415 ms to 575 ms, without averaging across segments from the same condition, to preserve by-subject and by-item variance. Fixed effects included relatedness and position, while ROI and sequence were co-variates. Participants and Items were included as random effects in this single-trial analysis. The model-fitting procedure resembled the behavioral analyses. Fig 4 displays the mean voltage amplitudes for the entire epoch of 800 ms for each condition and for two factors (Relatedness and Position) in the central posterior regions within the selected N400 time window.

**Fig 5:**
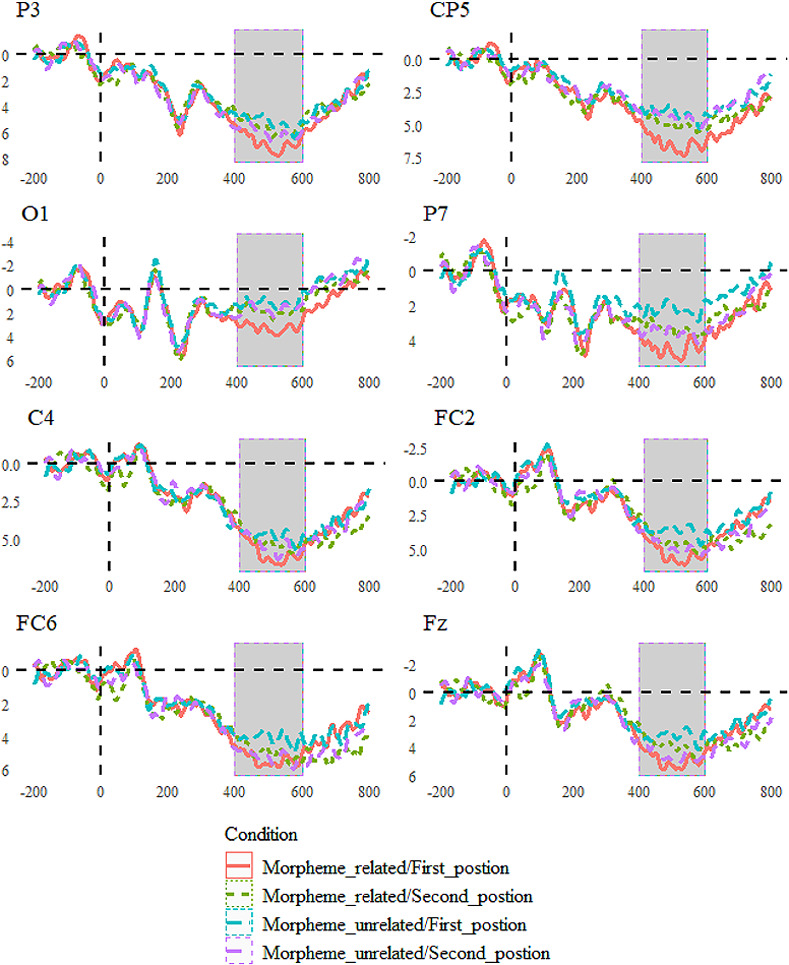
Voltage amplitudes by condition over time for each individual electrode. ROIs were included in the analysis of the long-lag picture naming task (n = 28). The time window of interest from 415 ms to 575 ms is highlighted in grey. Negativity is plotted up.

The model with the best fit was amplitude ~ relatedness * position + ROI + sequence + (1 | participant) + (1 | item) (see S3 Table). The Participant and Item’s random slope could not make the model converge, so they were not included in the current mode. This model yielded a main effect for *relatedness* with by-subject and by-item random intercept. The difference in amplitude between targets in the morphologically related and unrelated conditions was significant (*β* = -2.22, *SE* = 0.698, *t* = -3.185, *p* = 0.00285). The difference in amplitude between the first and second position conditions was also significant (*β* = -1.497, *SE* = 0.093, *t* = -16.107, *p* < 0.001). There was an interaction between *relatedness* and *position* (*β* = -2.09, *SE* = 0.1356, *t* = -15.419, *p* < 0.001). The sequence effect was significant with *β =* 0.414, *SE* = 0.455, *t* = 9.106, and *p* < 0.001. Grand-average ERPs for the four conditions are shown in Fig 5 for visualization of the individual channels included in this analysis.

## Discussion

This study investigated morphological encoding in Chinese disyllabic compound words and their storage in our mental lexicon. In a long-lag priming experiment, which included a substantial delay between primes and targets, thirty-six native Mandarin Chinese speakers were asked to name pictures on a screen. Behavioral data and electrophysiological data were collected and then analyzed.

The behavioral results were surprising and went against earlier findings in Indo-European languages, showing that the target picture naming was *not* facilitated by morphologically related primes in both first and second positions. Note that the first-position condition did elicit shorter RTs than the second-position condition. This suggests that morphological priming for individual constituents in producing Mandarin Chinese disyllabic compound words at the behavioral level does not occur.

The results reported here have implications for the two models of lexical representation discussed in the Introduction. According to Levelt and colleagues’ [3] model, compounds are stored in their decomposed form at the lexeme level. Consequently, the targets should be able to retrieve their lexeme representations faster if they had already encountered them in primed pictures (in contrast to unrelated pictures). Contrastingly, according to the single-stage model of lexical representation [1], compounds are stored in their full listing format at the lexical level. In other words, the whole word form is retrieved when naming the pictures instead of the individual constituents. Thus, when naming individual constituents, participants would have to retrieve these again, which predicts that the naming latencies should be the same when naming morpheme-related prime pictures in contrast to unrelated condition prime pictures. In line with this prediction, the results from the present experiment revealed that the naming latencies between morpheme-related and morpheme-unrelated conditions make no significant difference. Additionally, our results seem to support the results shown by others [10, 12].

However, in the ERP analyses, morpheme-related prime stimuli did elicit a reduced N400 at the central posterior regions when compared to morpheme-unrelated prime stimuli in the 415 ms– 575 ms time window, which is in line with previous results [21, 37] at both the temporal and spatial levels. The reduced negativity can be interpreted as an N400 effect, as the N400 is sensitive to morphological processing (21,29). This result suggests that the morphological priming effect occurs at the morphological encoding level and supports a decomposed model.

Previous research [21, 37] suggested that the outcomes stemmed from a genuine morphological process. Similarly, we claim that the current effects cannot be explained by a semantic or phonological relation between primes and targets. This is because semantic and phonological effects usually do not last beyond the temporal gap between prime and target in a long-lag paradigm [18, 51].

Furthermore, the timing of our ERP priming effects aligns well with the timeline for morphological encoding, as opposed to other cognitive phases like conceptual preparation or lemma retrieval [34]. Indefrey and Levelt proposed that morphological encoding, the primary phase in word form encoding, starts around 330 ms post-picture presentation. The initiation of the N400 effect in our study closely mirrors this predicted onset [34]. These projections are based on a response latency of 600 ms. Adjusting the onset of morphological encoding to correspond with a response latency of 750 ms– 800 ms in the present study, our observed average reaction time should yield a rough estimate of 413 ms– 440 ms for the initiation of morphological encoding. Interestingly, this estimation is almost identical to the onset of the N400 effects in the present study. Consequently, the N400 effects support the hypothesis that our morphological priming effect occurs and originates at the word-form level.

One question remains, however: how can we account for the divergence between the ERP findings and the absence of RT effects in the present study? In previous work [10, 12], a potential explanation was proposed, namely that the absence of a morpheme priming effect in Mandarin Chinese might be due to cross-linguistic differences. The involvement of morphological representations may not be significant in languages characterized by straightforward morphology, such as Mandarin Chinese. Conversely, these representations will likely be more prominent in languages featuring intricate morphology, like Dutch.

Another possible explanation might relate to the long-lag paradigm, which may have lacked the power to pick up on any fine-grained modulation in the Chinese morphological encoding process. Potential future research could focus on investigating the implicit RT results. However, EEG data are sensitive enough to capture these differences because ERPs offer a higher temporal resolution that enables more direct observation of cognitive processes, even before or without an explicit response [25]. EEG can provide more information on the current research questions regardless of the implicit outcome of naming latencies.

In addition, we did not control for transparency in the present study. On the one hand, opaque words are not common in Mandarin; on the other hand, many opaque words are mono-morphemic compounds (like 沙发 /sha1fa1/ “sofa”) in Mandarin. However, according to Koester and Schiller [21], Zwitserlood et al. [18, 19], and Verdonschot et al. [23], the opaque and transparent words elicited no significant difference in morphological priming. We believe more research could be done regarding this aspect of Mandarin.

The results in the present study suggest that morphological priming for constituents is present in the morphological encoding process of Chinese disyllabic compound words, even though the naming latencies between morphologically related and unrelated conditions yielded no difference. However, the morphological involvement is not as strong as in Dutch. Chinese disyllabic compounds are stored with two separate constituents instead of the whole word representation. This suggestion aligns with a decomposed storage model [3, 15, 35] rather than a single-stage model [1, 10, 12]. Then, given the presence of morphological encoding, the morphological priming effect originates in the word form level according to the temporal course of the N400 effect.

The repeated picture presentation across two blocks lead to reduced picture naming latencies in this experiment, according to the statistical results in both naming latencies and ERP analyses. This effect is consistent and comparable to previous findings [18, 19]. Importantly, the block effect did not interact with manipulating the variable of primary interest, i.e., Prime type. Therefore, it is suggested that the general facilitation across blocks is independent of linguistics processes. This repetition effect may, for example, reflect the more efficient visual processing or recognition of the pictures.

Regarding the position effect, the present results from RTs showed that the naming latencies for the first position were shorter than the second, which aligns with our expectations, even though this difference is not significant. In the ERP results, the significant effect of position and interaction between relatedness and position showed that the first and second constituents were processed differently. A larger reduced N400 was found for the first than the second position. These findings support the claim that both positions have priming effects, but the priming effect is larger for the first position. The significant difference between the first and the second constituents supported the serial planning assumption and the modifier constituent facilitation effect. However, the challenge of understanding how the head constituent influences compound production, as observed in [15], remains unsolved. This influence could be either facilitatory or inhibitory in nature. Further research is necessary to shed light on this issue.

In addition, there were both free and bound morphemes in the stimuli of the present study. Bound and free morphemes could cause differences in the processing of Chinese compounds. There might be a possibility that compounds with different morpheme-type combinations are processed differently, which is worth exploring. This could also be why our behavioral data did not have an effect and only the first position morphological priming effect was found in the ERP analysis. We did a post-hoc analysis regarding two types of morphemes, but no significant statistics results were found (*p* > 0.05). This might be because we had balanced the items for two morpheme types. More research can be done on this aspect in the future.

To conclude, in the present long-lag priming experiment, picture naming was behaviorally not facilitated by the production of morphologically related compound words. However, morphological priming for individual constituents was present from an N400 effect in the central posterior region in the ERP analysis, especially concerning the priming of the first constituent. The N400 effect presented in the process appears to originate at a word form level corresponding to the temporal estimate of morphological encoding, and two individual constituents are both activated and processed during morphological encoding.

## Supporting information

S1 Table Experimental stimuli. (PDF)

S2 Table Specification of best-fit model for RTs (ms) for n = 32. (PDF)

S3 Table Specification of best-fit model for voltage amplitudes (microvolts) for n = 28. (PDF)

S1 Fig EEG montage. 10/20 system—32 channel montage from BioSemi.
(PDF)
