# Supplementary material for: Morphological encoding in language production: Electrophysiological evidence from Mandarin Chinese compound words
Source: PLoS One. 2024 Oct 2;19(10):e0310816. doi: 10.1371/journal.pone.0310816 (PMC11446431; doi:10.1371/journal.pone.0310816)
Supplement: S1 Table — (PDF) [file pone.0310816.s001.pdf]

S1 Table. Experimental stimuli

Target	Pinyin	Meaning	Morpheme-related distractor	Pinyin morpheme-related distractor	Meaning morpheme-related distractor	Morpheme-unrelated distractor	Pinyin morpheme-unrelated distractor	Meaning morpheme-unrelated distractor
山	shan1	mountain	山羊	shan1yang2	goat	飞机	fei1ji1	airplane
鸟	niao3	bird	鸟巢	niao3chao2	bird nest	蜡烛	la4zhu2	candle
脚	jiao3	foot	脚印	jiao3yin4	footprint	熊猫	xiong2mao1	panda
牙	ya2	tooth	牙刷	ya2shua1	toothbrush	南瓜	nan2gua1	pumpkin
竹	zhu2	bamboo	竹简	zhu2jian3	bamboo slip	蜂蜜	feng1mi4	honey
手	shou3	hand	手机	shou3ji1	mobile phone	拼图	pin1tu2	puzzle
眼	yan3	eye	眼罩	yan3zhao4	eye mask	钢琴	gang1qin2	piano
鱼	yu2	fish	鱼竿	yu2gan1	fishing rod	树桩	shu4zhuang1	tree stump
鹿	lu4	deer	鹿角	lu4jiao3	antler	领带	ling3dai4	tie
灯	deng1	lamp	灯塔	deng1ta3	lighthouse	孔雀	kong3que4	peacock
信	xin4	letter	信封	xin4feng1	envelope	香蕉	xiang1jiao1	banana
雨	yu3	rain	雨伞	yu3san3	umbrella	皇冠	huang2guan4	crown
雪	xue3	snow	雪人	xue3ren2	snowman	青蛙	qing1wa1	frog
月	yue4	moon	月饼	yue4bing3	moon cake	翅膀	chi4bang3	wing
旗	qi2	flag	旗袍	qi2pao2	cheongsam	秋千	qiu1qian1	swing
书	shu1	book	书架	shu1jia4	bookshelf	蘑菇	mo2gu1	mushroom
轮	lun2	wheel	轮胎	lun2tai1	tire	彩虹	cai3hong2	rainbow
鼠	shu3	Mouse (animal)	鼠标	shu3biao1	mouse (computer)	钮扣	niu3kou4	button
石	shi2	stone	石榴	shi2liu2	pomegranate	樱桃	ying1tao2	cherry
草	cao3	grass	草莓	cao3mei2	strawberry	电话	dian4hua4	telephone
车	che1	car	风车	feng1che1	windmill	羽毛	yu3mao2	feather
刀	dao1	knife	剪刀	jian4dao1	scissors	玉米	yu4mi3	corn
针	zhen1	needle	别针	bie2zhen1	safety pin	围巾	wei2jin2	scarf
脑	nao3	brain	电脑	dian4nao3	computer	钥匙	yao4shi	key
杯	bei1	cup	奖杯	jiang4bei1	trophy	毛巾	mao2jin1	towel
盘	pan2	plate	键盘	jian4pan2	keyboard	苹果	ping2guo3	apple
线	xian4	wire	电线	dian4xian4	electric wire	邮票	you2piao4	stamp
球	qiu2	ball	气球	qi4qiu2	balloon	老虎	lao3hu3	tiger
船	chuan2	boat	飞船	fei2chuan2	spaceship	骆驼	luo4tuo	camel
花	hua1	flower	烟花	yan1hua1	fireworks	喷泉	pen1quan2	fountain
包	bao1	bag	面包	mian4ba1o	bread	天鹅	tian1e2	swan
梯	ti1	ladder	滑梯	hua2ti1	slide	洋葱	yang2cong1	onion
绳	sheng2	rope	跳绳	tiao4sheng2	jump rope	戒指	jie4zhi3	ring
扇	shan4	fan	电扇	dian4shan4	electric fan	磁带	ci2dai4	cassette tape
箭	jian4	arrow	火箭	huo3jian4	rocket	菠萝	bo1luo2	pineapple
镜	jing4	mirror	泳镜	yong3jing4	goggles	乌龟	wu1gui1	turtle
龙	long2	dragon	恐龙	kong3long2	dinosaur	口红	kou3hong2	lipstick
桶	tong3	bucket	马桶	ma3tong3	toilet	面具	mian4ju4	mask
钉	ding1	nail	耳钉	er3ding1	earring	烟斗	yan1dou3	pipe
牛	niu2	cow	蜗牛	wo1niu2	snail	卡车	ka3che1	truck
