# Supplementary material for: Morphological encoding in language production: Electrophysiological evidence from Mandarin Chinese compound words
Source: PLoS One. 2024 Oct 2;19(10):e0310816. doi: 10.1371/journal.pone.0310816 (PMC11446431; doi:10.1371/journal.pone.0310816)
Supplement: S2 Table — (PDF) [file pone.0310816.s002.pdf]

S2 Table: Specification of best-fit model for RTs (ms) for n = 32.

**Formula: RT ~ Relatedness + Position + Sequence + (1 | Subject) + (1 | Item)**				
Fixed effects	**Estimate**	**95%CI**	**t-value**	**p-value**
(Intercept)	1.32	[1.075, 1.605]	25.92	<0.001 ***
Relatedness: Morpheme unrelated	0.012	[-0.203, 0.224]	1.16	0.247
Position: Second position	-0.051	[-0.28, 0.235]	-1.14	0.254
Sequence	-0.059	[-0.138, 0.2988]	5.69	<0.001 ***
**Random effects**				
σ^2	0.050			
τ00Item	0.005			
τ00Subject	0.006			
ICC	0.098			
NSubject	32			
NItem	40			
Observations	2560			
Marginal R^2	0.025			
Conditional R^2	0.200			
