# Supplementary material for: Morphological encoding in language production: Electrophysiological evidence from Mandarin Chinese compound words
Source: PLoS One. 2024 Oct 2;19(10):e0310816. doi: 10.1371/journal.pone.0310816 (PMC11446431; doi:10.1371/journal.pone.0310816)
Supplement: S3 Table — (PDF) [file pone.0310816.s003.pdf]

S3 Table: Specification of best-fit model for voltage amplitudes (microvolts) for n = 28.

**Formula: Amplitude ~ Relatedness * Position + ROI + Sequence + (1 | Participant) + (1 | ITEM)**				
**Fixed effects**	**Estimate**	**95%CI**	**t-value**	**p-value**
(Intercept)	6.05	[3.696, 8.179]	5.143	< 0.001***
Relatedness: Morpheme unrelated	-2.224	[-3.570, -0.805]	-3.185	0.000285
Position: Second position	-1.497	[-1.689, -1.309]	-16.107	< 0.001***
ROI: left posterior	-1.209	[-1.307, -1.109]	-23.150	<0.001***
Sequence	0.414	[0.325, 0.501]	9.106	<0.001 ***
Relatedness Morpheme unrelated *Position Second	2.092	[1.840, 2.360]	15.419	<0.001 ***
**Random effects**				
σ^2	311.798			
τ00Item	4.809			
τ00Subject	31.862			
ICC	0.014			
NSubject	28			
NItem	40			
Observations	610080			
Marginal R^2	0.003			
Conditional R^2	0.108			
