# Supplementary material for: Morphological encoding in language production: Electrophysiological evidence from Mandarin Chinese compound words
Source: PLoS One. 2024 Oct 2;19(10):e0310816. doi: 10.1371/journal.pone.0310816 (PMC11446431; doi:10.1371/journal.pone.0310816)

S1 Fig: EEG montage. 10 / 20 32-channel montage from BioSemi (www.biosemi.com/headcap.htm).

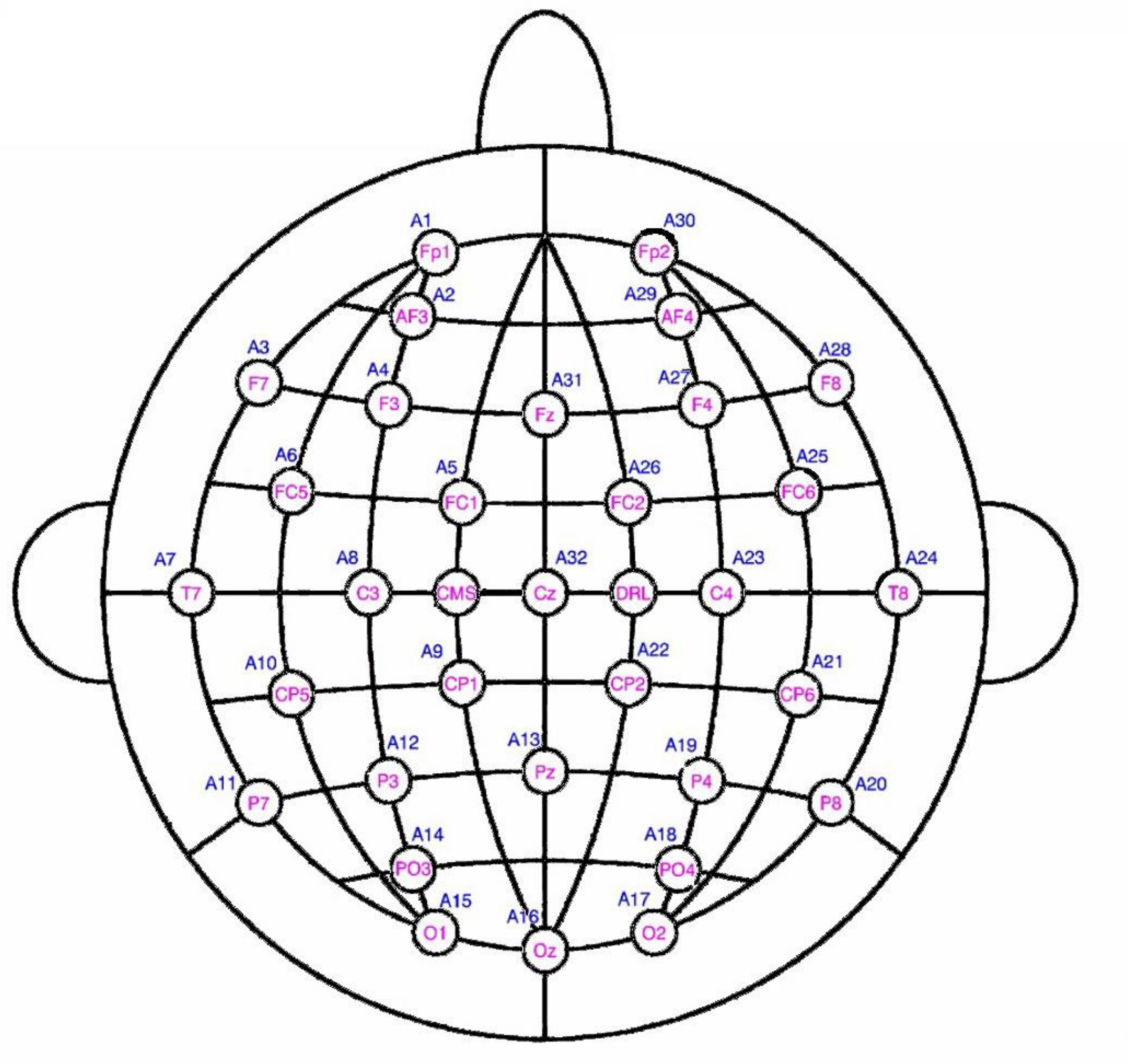

Supplement: S1 Fig — 10/20 system—32 channel montage from BioSemi. (PDF) [file pone.0310816.s004.pdf]
